# Analysis of Albania's diplomatic relations with the Soviet Union during the years 1948-1959

**DOI:** 10.12688/f1000research.126497.2

**Published:** 2024-02-09

**Authors:** Manjola Xhaferri, mirela tase

**Affiliations:** 1 Department of Political Science, Aleksander Moisiu University, Durres, Albania, 2001, Albania; 2 Department of Tourism, Aleksander Moisiu University, Durres, Albania, 1001, Albania

**Keywords:** Relations, diplomacy, Soviet Union, Analysis, Albania

## Abstract

**Background:**

This paper aims to shed light on a very intense period of relations between Albania and the Soviet Union (USSR), focusing on the period from 1948 with the intensification of Albania’s relations with the USSR until the termination of diplomatic relations in 1959. The paper will overview the USSR’s relationship with Albania and its behaviour.

**Methods:**

The paper analyses a specific period between 1948 and 1959 as a case study. The analysis is conducted on context, actors, and outcomes. The research aims to identify key indicators and the implications of the relationship in both domestic and international contexts. The data sources include various materials such as interviews, films, newspapers, and books. The researchers have requested permission to use the Archival resources of the Albanian Central State Archives (AQSH), where they have made some non-state secret resources accessible. However, some of these resources can only be accessed within the archive, but the researchers have been permitted to use them.

**Findings:**

The facts show that the relations between Albania and the USSR benefitted Albania more. particularly with economic support from the USSR, which helped Albania emerge from the crisis and build the so-called ‘socialist state.’ Albania took its place in the international arena through these relations and contributions.

**Conclusions:**

The study, regardless of the issues it addresses, does not aim to exhaust the paper’s topic but to contribute to the objective analysis and to have some impact on the continuation and deepening of the treatment of this topic. Albania’s relations with the Soviet Union are still not fully revealed as long as many documents found in Russian archives are not available for study. But further, numerous documents show that this could be the ‘best cover’ to safeguard the interests and personal power of the leadership.

## Introduction

This paper corresponds to one of the most critical periods of history regarding the national and international policy of the Albanian state, especially the isolated political and state cooperation of Albania with the Soviet Union. Albanian-Soviet relations were significant, as they determined the orientation of Albanian domestic policy and the foreign behavior Albania had to maintain in the international arena, especially in relations with Western allies and neighbors.

This paper refers specifically to the period from 1948, when the Albanian-Soviet relations took their official form and capacity, until 1959, when these relations were broken. Many internal and external political situations have been touched upon to explain better the reasons and causes of establishing these relations, which go beyond the defined time limits of our study. The primary purpose of this paper is to analyze the conditions in which these relations are established and their unusual bilateral cooperation in the political, economic, international, and military fields. What is very important to include are the moments of establishing the Albanian-Soviet relations, their development, and completion in the most peaceful way possible. The paper aims to approach this relationship from a new study perspective to understand its true nature. In this paper, we will try to clarify some research questions that orient us toward clarification by understanding the truth of the facts. Specifically, we will examine:

• How much space was devoted to Albania in the international communist movement?
• How important was Albania to the Soviet Union?

There was not only the economic growth in the years 1950-1960 to consider, but what was an unusual phenomenon for the poor Albanian people, coming from a backward economic tradition, was the social development of the Albanian society, where we will focus on distinct areas: housing, employment, health, the fight against illiteracy, etc. What we noticed in this study is that education, as well as culture, increased the trust in the communist government in Albania, which was part of the perfect implementation of the Soviet model, not only politically but also socially. The whole ‘Sovietization’ of the Albanian state and society was used by the Union of Soviet Socialist Republics (USSR), as with all countries of the former Eastern bloc, to realize its hegemony in the international arena. In conclusion, I would summarize with the expression, “Stalin was the political muse for Enver Hoxha, this worship that he had continued until the end of his life and refused to be de-Stalinized. This turned out to be fatal in relations with the Soviet Union and all other countries and ended in the greatest isolation of Albania until the fall of the regime in 1990”. ^1^

## Research questions and hypotheses of the paper

### Research questions of the study

Albania’s relationship with the Soviet Union has been very complex. By analyzing the documents from the relevant period and examining the evidence, a series of research questions can be formulated to determine whether Albania’s relationship with the superpower has been presented truthfully to the public or if there is more to the story than what currently appears. The research questions to address this are:

a. Has Albania been entirely dependent on the Soviet Union?
b. Was Albania really in the spotlight of the Soviet Union as the idea was created in Albania over the years?
c. Was it possible for Enver Hoxha to behave differently with the Soviet Union, and what has been Albania’s strong point in relations with the Soviet Union?
d. What was hidden in the telegrams, radiograms, and secret letters exchanged between the ambassadors, the officials of the delegations, and even those of the great leaders of both countries? What was their language? These sources were accessed only by the Central State Archive source of the Foreign Ministry. Some of the items were restricted as they were considered state secrets, but we hope that 50 years after Hoxha’s passing away, these archives will open and give us access to use them. It will be a challenge for us to read more secret documents required to continue this study in the future.
e. Did Khrushchev plan to attack Albania, and could he do so, considering the country’s geographical position?

These and other questions will be answered in this paper. The paper aims to prove whether the relationship with this superpower has been as it has been presented to us so far. Albania was essential in relations with the Soviet Union, or things were not as they seemed.

Our analysis will be focused on several aspects, namely: a) the impact of the relations between Albania and the Soviet Union, b) the individual cults of leadership in both countries, c) the ideological divergences and their effect on bilateral relations, and d) the economic dependence resulting from the relations between Albania and the Soviet Union.

### Hypotheses

This study offers three hypotheses that address the critical problem. The main concern of the hypotheses is the clarification of this bilateral relationship, which has had a lot of light and shade obscuring it. This paper emphasized giving an unbiased overview of the situation, considering the ideological load of the documentary sources of that period. We tried, through hypotheses, to provide an assessment and undoubtedly a clearer insight into the relations with the Soviet Union. According to our judgment, Albania was an unimportant country for the Soviet Union. Enver Hoxha benefited from this relation to building the socialist state and giving it a name in the international arena, not only for himself as the Albania head but also for Albania as a whole. In each section, this study tries to show the integrity of these hypotheses.

***The paper’s primary hypothesis*** is that the Soviet Union did not have a significant geopolitical or economic interest in Albania, as previously assumed. Instead, the Soviet Union and Albania drew closer mainly due to the breakdown of relations between the Soviet Union and Yugoslavia and Albania with the latter. Without international economic and political support, Albania turned to the Soviet Union for economic and political assistance to fill the void created by the split with Yugoslavia.

***The paper’s second hypothesis*** is that Albania’s relationship with the Soviet Union was based on dependence and preferences. Despite having the same ideology, Albania’s relations with the Soviet Union were dominated by pressure, blackmail, and obedience. Therefore, their relationship reflected the relationship between powerful countries.

***The third hypothesis*** deals with the Yugoslavs, as Albania was never a priority for the Soviet Union. The country was considered depending on the fluctuations of Soviet-Yugoslav relations, which remained unchanged during both Stalin’s and Khrushchev’s time. Therefore, Albania’s significance was not determined by the strength, position, and close ties between Yugoslavia and the Soviet Union. So, Albania was not measured by the strength, position, and close connections between Yugoslavia and the Soviet Union. In some cases, Enver Hoxha claimed that the connection between his country and the Soviet Union was strong and inseparable. This claim was mainly made during the break-up of the Yugoslavia-Soviet Union relations. But this showed the opposite; it was just a facade, as Hoxha exploited this fact for his political interests, and even the fanaticism he expressed towards Stalinism was an excuse for the fear that he had for the longevity of his power and of being replaced by anyone including another Albanian leader who might come to power.

## Methods

The research model on which the paper is based is that of the case study, focusing on the context of international relations. This paper follows a combined research strategy as outlined below:

### Evaluative research

‐ Evaluation of the period (1948–1959) through identifying key indicators, their values, and their impact on the analysis of the case study.
‐ Evaluation of the implications of the relationship in the domestic and international context through the analysis of the behavior of the actors, their discourse, and strategic documents.

This model is mainly used to answer the research questions posed in the previous section. The basis of the research work was the research and study of documents and testimonies of the protagonists as local and foreign literature about the problems related to the Eastern bloc in the years 1948–1959 (Some protagonists of the drives have died and, some are in the form of memories that we have quoted in our work). In terms of literature, world-renowned works examined have described and analyzed the model of the Stalinist dictatorship in Albania and the behavior of the dictator Enver Hoxha in the international arena. These are works translated by the Albanian National Library and the Central State Archives. Also, a special place involves the documents of the central committee of the Albanian Labor Party, as well as the works of Enver Hoxha, ^2^^–^^5^ which include valuable documents. As we have mentioned in the methods session, for the realization of this work, in addition to publications by different authors and periodicals from the period of communism, we also used archival sources, which are not available to the public in digital form. The only way to utilize it is to study on the premises of the central archive of the state. The sources used in the archives belong to the 1953–1959 years. We have integrated all the sources in the paper, mainly in the history of Albania-Soviet Union relations. Using the same sources from other researchers below gives us a way to identify them.

The first archival source of the Central State Archive is the manuscript of the Ministry of Foreign Affairs entitled: “History of Relations between Albania and the Soviet Union,” file number I-8, entitled New Albania, number pages 48, the year 1953. This archival document contains several pages devoted to the beginnings of Diplomatic Relations between Albania and the Soviet Union and the continuation until their final breakdown. These documents list the ‘good things’ that the Soviet Union has done for Albania. “The leaders of the Soviet people, Lenin and Stalin, have repeatedly exposed the aggressive policy of the imperialist powers for the fragmentation of the world on the one hand and Albania on the other.

The result of this source is that the Soviet Union has supported Albania in several political and financial moments. Great political support was when the leader of the Bolshevik Revolution, Lenin, was on Albania’s side, opposing the bargains of the great powers at the Conference of Ambassadors in London in 1913. Another support of the Soviet Union, vis-à-vis Albania, was the financial one during the time of Stalin or later to Nikita Khrushchev. So Albania, even though it was not the main focus of the policies of the Soviet Union, was constantly helped financially by the three leaders of the Soviet Union, Lenin, Stalin, and Khrushchev. They used Albania as a strategic place for their interests in the Balkans.

The second archival source of the Central State Archives is the manuscript of the Ministry of Foreign Affairs entitled: “History of Relations between Albania and the Soviet Union,” with file number I-19, entitled XX Congress of the Communist Party of Soviet Union, number of pages 118, the year 1956. This archival document contains the proceedings of the 20th Congress of the Communist Party in BS. For the first time, Khrushchev openly criticizes Stalin’s cult of the individual.

The result of this source is that 20th Congress bells rang in the ears of the Albanian leader as an alarm. The influence of the 20th Congress of the Communist Party of the Soviet Union was huge. We cannot say what Enver Hoxha’s reaction was to Khrushchev’s statements, but what he did publicly was that first, he did not hesitate to come out against Stalin’s cult of the individual.

The third archival source of the Central State Archives is a manuscript of the Ministry of Foreign Affairs entitled: “History of Relations between Albania and the Soviet Union,” with file number I-6 entitled “Letter sent by Enver Hoxha to the Central Committee of the Communist Party of the Soviet Union and specifically to Nikita Khrushchev on April 14, 1958”, page number 3, the year 1958. Enver Hoxha wrote enthusiastically about the decision taken by the Central Committee of the Communist Party of the Soviet Union regarding the non-sending of the Soviet delegation to the Seventh Congress of the League of Communists of Yugoslavia.

The result of this source is that relations with the Soviet Union after Stalin’s death could never function independently of Yugoslavia’s relations with the Soviet Union.

The fourth archival source of the Central State Archives was a manuscript of the Ministry of Foreign Affairs entitled: “History of Relations between Albania and the Soviet Union,” with file number I-92, entitled “Nikita Khrushchev’s visit in May 1959”, page number 85, 1959 year.

During his visit to Albania, Nikita Khrushchev gave a speech at a large rally with the workers of the “Stalin” textile factory, a gift from the Soviet government to Albania.

The result of this source is that Khrushchev’s visit was not as successful as the Communist Party leader Enver Hoxha defined it. Khrushchev addressed the government with critical notes regarding the country’s development, where he asked for an account of how the financing made by the Soviet Union invested, where Albania should be a country many times more developed than with all these natural resources that God had given them.

### Data collection

The sources for the documents used in this study are available in the Archive of the Ministry of Foreign Affairs ( https://www.arkiva.gov.al). Access to the records is only available as hard copies. All sources in hard copy are referenced or cited in the manuscript.

Most of the resources are provided by the State Archives. All sources used in the central archive of the state are referred to in the manuscript. To use any source in the archives, authorization is required through a request for the topic of the resources you need to operate. After the approval of the authorization, the archive staff will make available the available resources. The search for sources was done in cooperation with the archive staff, who advised us to use the files related to the documents about the diplomatic relations between Albania and the Soviet Union. In the review procedure for previously used sources, the researcher, through a request, must provide arguments for previously used documents and what conclusion will be drawn from the review.

Data collection and the study’s realization include 2018–2020. All the resources are provided to us in the Albanian language, where, without a doubt, the resources in foreign languages have been translated by the translation department within the State Archives.

The criteria for the documents to be included in the work is that the line of relations must be maintained without accusations and allusions, taking into account the form of the regime at that time, the way the communist system built diplomatic relations, without nationalistic spirit, or unilateral political influences, thus preserve the professional ethics of the researcher (historian).

### Comparative research

Comparative research consists of ascertaining the similarities and differences between different phenomena, in this case, to better understand and interpret the behaviors of the states (Albania and the Soviet Union) and their specific policies, in this case, regarding the relations between the two countries. Comparisons between fields, factors, actors, or even countries or organizations can be made. In terms of time, they can be at other times, diachronically or synchronously, at the same time. Comparative studies can be intra-state, inter-regional, or global. The empirical approach occupies a special place in the methodology of scientific research in political sciences. In this case, she tries to interpret the reality of international politics as objectively as possible throughout the years under study through observations, experiences, instruments, and specific scientific methods.

The case analysis of the Yugoslav influence in relations with the Soviet Union will remain a case study in international relations. Concerning the Yugoslavs, Albania has never been a priority for the Soviet Union. It would have to be treated depending on the ups and downs of Soviet Union-Yugoslavia relations. This did not change both under Stalin and Khrushchev. Disputes within the communist bloc, such as those between Yugoslavia and the Soviet Union or Albania and Yugoslavia and the Soviet Union, showed that there could be divergence even when countries follow the same strategy and ideology. The reason for these divergences in these cases has been sovereignty as an essential issue of national character. Regarding Albania, the facts show that the national interest fuelled Enver Hoxha’s ego and struggle for power. He used it for his own sake, to maintain his jurisdiction.

### Limitations

The methodological limitations of the paper relate to the difficulty of balancing the documentation available to us due to the different perspectives between the parties and the documentation of Albanian-Russian relations. Russia continues to be quite conservative in publishing documentation for this period. The Albanian materials we have at our disposal and belong to this period (1948–1959) have ideological solid and propaganda loads. Finding materials and resources has not been difficult because academic publications on this topic are numerous and have abundant material. I would emphasize mainly the sources in the Central State Archive, where there are thousands of manuscripts, such as personal letters or diplomatic agreements between the two countries. Other limitations include the volume of domestic documentation, the data available, and the difficulties in selecting the most appropriate statements or documents. It is also worth noting the limited number of analyses by third parties due to the small weight of Albania-Soviet Union relations, international relations, etc. To minimize the consequences of such limitations, the paper focuses on using analysis in the context of this period as a case study, selecting three levels (context-actors-outcomes) to help place the paper in an internal coherence. The actors are the Soviet Union-Albania-Yugoslavia. It also focuses on a defined number of indicators of the implications of bilateral relations, such as stakeholder lectures and critical strategic documents. The institution has provided the primary documents in the state’s central archive. For the sources used in these archives, authorization requires a request for the topic of the resources. The search for sources was done in cooperation with the archive staff, who advised us to use the files related to the documents about the diplomatic relations between Albania and the Soviet Union—the time limit for this purpose (1948–1959). As we emphasized above, a special place was occupied by the documents of the central committee of the Work Party of Albania and the works of Enver Hoxha, which include valuable documents. Although with a high ideological charge, these sources have given this paper a picture of how diplomatic relations were built between the countries of the former communist bloc. More details are included in the methodology section of the abstract.

## Overview of the history of Albania-Soviet Union relations

The relations of the Albanian people with the peoples of Eastern Europe began with the Russo-Turkish War of 1768–1774, a war that started due to France’s encouragement to the Turks. ^6^ According to a document of the Central Archive of the Albanian State, it turns out that in the fight, the Turkish sailors fought many Albanians who came from Albanian lands, which at that time were under the occupation of the Ottoman Empire, referring to a document of 1953. ^6^ These Albanian fighters were decorated with medals of bravery for the contribution given by the Russian state. ^6^ According to the documents discovered in the archives of the Albanian state, it turns out that the Albanian volunteers took part in many battles alongside the Russians against the Turks. Enver Hoxha, in the early documents of the communist era, finds evidence in these times for the melding of Russian-Albanian friendship. ^6^

According to these documents, during the Ottoman occupation, many Albanians, finding it impossible to live in subjugation, emigrated. Some settled in Russia. Several others settled in several villages in the Zaporizhzhia region of Ukraine. ^7^ According to many documents in the Russian archives referred to by Russian scholars who have dealt with this topic, it turns out that these Albanian fighters were from the south of Albania, mainly from the province of Himara. ^8^ In one of his works, Shaqir Vukaj, ^9^ the current Albanian ambassador to Moscow and historian, writes about the Moscow myth. According to him, although Albanian-Russian relations have been little studied, it seems that in the –fifteenth to eighteenth centuries, the myth of Moscow was raised in Albania, primarily as a savior of Christians who suffered under the Ottoman yoke. Some Albanian bishops went to Moscow not only to ask for Russia’s interests in the Balkans early, but it was Peter the Great who, with his call in 1711, made Russia directly involved in Balkan developments. ^8^

Later, Lenin, in his 1915 work entitled ‘Socialism and War,’ ^7^ opposing the argument that war was not made to take but to defend the homeland, wrote, among other things: “Only hypocrites hide this. England plundered the German colonies, and Turkey, Russia, Galicia, and Turkey, and France are looking for Alsace Lorraine and even the left bank of the Rhine; with Italy, a treaty has been concluded on the division of the spoils (of Albania and Asia Minor)”. ^7^

In archival documents about the years of dictatorship, all this is interpreted as early support of Russia to Albania. These documents, mainly from the 1950s, list the ‘benefits’ that the Soviet Union has done for Albania; “The leaders of the Soviet people and all mankind, Lenin and Stalin, have constantly unmasked the aggressive policy of the imperialist powers to divide”. ^7^

Enver Hoxha dedicated the liberation of Albania primarily to the war of the people of the Soviet Union, the Soviet army, and the Bolshevik party. At least, this is what the leader of the Albanian communists himself says in one of the documents of the central archive of the Albanian state. ^7^

However, as enough has been written about this topic, even according to the scholar William Griffith, Hoxha was placed at the head of the Albanian communists, thanks to the Yugoslav contribution on November 8, 1941. ^10^ With the help of the British and Yugoslavs, the Albanian communists finally managed to win a long and successful partisan war. ^11^ Even though Enver Hoxha, in his writings, tried to make the friendship with the Soviets clear as early as possible, the facts show that Albania until 1948 remained a Yugoslav satellite more than a Soviet one. As a country, Albania did not attend Conference meetings and was represented by Yugoslavia instead. However, like all communists who came to power with their forces, the Albanian communist leadership pursued the extreme left ‘Stalinist’ policy both inside and outside the country. But the Yugoslavs were allowed to negotiate with the Albanians, perhaps more than Moscow wanted. We recall creating a sizeable federal state, including Albania, which Stalin opposed. ^1^ From the first days after the liberation, Albania’s foreign policy-oriented created diplomatic relations with the countries that aligned themselves in honour of the anti-fascist war against the Nazi-fascist bloc. The country with its first diplomatic relations was the Soviet Union, from which Albania did not break away until the death of Stalin. The economic period of domination of Albania by the Soviet Union began in September 1948, when they signed the first economic agreement. It was finalized during Enver Hoxha’s visit to Moscow in 1949. After the signing of this agreement, other Soviet Union (USSR) satellites also started to support Albania. ^10^ By 1949, 38 percent of the Albanian state’s internal revenue came from loans as aid grants from the Soviet Union blocked several countries. ^10^ Meanwhile, an intensive program, especially in industry, ^10^ began under Soviet Union leadership. During 1951–1955, periodic industrial production increased threefold. ^10^ But in 1953, Stalin’s death and Khrushchev’s approach to Belgrade in 1955 threatened Enver Hoxha with de-Stalinization and the emergence of Yugoslavia. It was led by reduced aid to Albania in the 1953–1955 years, both in machinery and other investments. ^10^

## Albania’s relations with the Soviet Union after Stalin’s death

The period of political and economic domination of Albania by the Soviet Union began in September 1948, when the first economic agreement was signed. It was finalized during Enver Hoxha’s visit to Moscow in 1949. In 1953, the death of Stalin and Khrushchev’s coming to power and his rapprochement with Belgrade in 1955 threatened the policy of Enver Hoxha’s relations with the Soviet Union. Although limited to a few meetings, Enver Hoxha’s relationship with Stalin seemed to be loyal, at least on the part of the Albanian leader. Enver Hoxha was a Stalinist follower and remained so until Stalin’s death. On March 5, 1953, Stalin died. His statue in the center of Skanderbeg Square, in the Albanian capital of Tirana, was turned into a place of pilgrimage. In his book ‘The Bend and Fall of Albanian Tyranny,’ Spartak Ngjela describes the dramatic atmosphere after Stalin’s death. As a witness, he says that for three consecutive days, the leadership block, the whole big house of the Central Committee, seemed shocked by the news of Stalin’s death. ^12^ The public lined up on that day in front of the statue to already honor the memory of Stalin so soon after his death. But life would go through the heavy weight of this significant loss to world communism. Although with the anxiety about the ideological and political line that the Soviet Union would follow in the future, Enver Hoxha would continue to praise this great country and call it a source of inspiration for further achievements. ^13^

## Disputes between Albania and the Soviet Union 1955–1957

The first disputes between the Soviet Union began after the death of Stalin and Nikita Khrushchev coming to power. This is because the new leader of the Soviet Union criticized the cult of Stalin and openly expressed the reform of the Soviet Union. From the documents already open in the Russian archives, it appears that the content of Nikita Khrushchev’s famous speech had already been decided at this meeting of the central committee. ^13^ The 20th Congress of the Communist Party of the Soviet Union convened on February 14, 1956, and lasted until the 25th of that month. ^13^ The most important event of this congress was the de-Stalinization of the Soviet Union, which the first secretary of the Soviet Communist Party, Nikita Khrushchev, announced in a long speech in which he analyzed his entire political career as Stalin’s leader. ^13^ The speech was held in an audience of people chosen and approved by Khrushchev himself. For the first time, Stalin was officially criticized for the cult of the individual and blamed for its consequences in the society of the Soviet Union. Khrushchev would begin his speech with the words: “Dear comrades, in the report of the party’s central committee for the twentieth congress and a considerable number of speeches delivered by delegates even during previous plenary sessions, much was told about the cult of the individual and its harmful consequences.” ^14^

Khrushchev further tried to explain that this report was not intended to judge and evaluate Stalin’s life and activity; he stressed that everyone worldwide recognizes Stalin’s role in implementing the socialist revolution, in the civil war, and in his efforts to build socialism in the Soviet Union. According to Khrushchev, the central committee analyzed what had happened to him after Stalin’s death. ^15^ This was the first speech regarding the content and feedback it brought. Never before had a senior Soviet leader spoken out against Stalin and his deeds. According to Khrushchev, Stalin enormously popularized himself as a great strategist, instilling in the minds of the people in all possible ways the version that all the victories won by the Soviet people in the great patriotic war were the result of Stalin’s manliness, bravery, genius, and no one else. ^16^

Enver Hoxha did not hesitate to give his opinion on this issue. The official organ of the Albanian Labor Party, *The People’s Voice, on April 14, 1956, was opened with the main article entitled ‘Marxism – Leninism’, which ^13^ teaches us that the people are the creators* of history. Enver Hoxha signed this article. In it, he expressed: “Stalin made mistakes that cost the Soviet people and the cause of socialism. The Communist Party of the Soviet Union rightly condemned the cult of the individual created for J.V. Stalin in the last years of his life activity and who did so much damage to the Soviet Union”. ^13^ It must be said that J.V. Stalin, at a time after the Communist Party of Soviet Union and the Soviet people achieved great victories that led to the triumph of socialism, began to put himself above the party and the people, to leave the masses. So, mistakes were made that cost the Soviet people a lot. and the question of socialism. ^13^

Enver Hoxha did not hesitate to criticize Khrushchev, his idol, as harshly. Stalin was Hoxha’s idol until his death, but for political reasons and personal interests, after the death of Stalin, Enver Hoxha temporarily criticized the figure of Stalin for maintaining relations with the Soviet Union, but this did not last long, and Albania broke relations with the Union. The Soviet Union oriented its foreign policy toward China. In the pages of *The People’s Voice*, Enver Hoxha writes further:

On the pages of the “Voice of the People” newspaper, Enver Hoxha writes further: “In the condemnation that the 20th Congress made of the cult of the individual, in the open and courageous criticism that it made of its damages, the Communist Party of the Soviet Union and all communist and labor parties of all countries conclude practical importance.” ^13^ Although Enver Hoxha tried to follow Khrushchev’s path, again the first sparks of distrust and paranoia brought about by the restoration of a friendship between the Soviet Union and Yugoslavia had begun to emerge. The effects of the 20th Congress of the BS KP were strongly felt in the Tirana conference of the Albania Social Party (ASP). Voices against the cult of the individual increased, and Enver Hoxha’s power was more endangered than ever. The effects of the 20th Congress of the Communist Party of the Soviet Union were strongly felt at the Tirana conference on the Socialist Party of Albania. Voices against the cult of the individual increased, and Enver Hoxha’s power was more endangered than ever. Hoxha interrupted his vacation in Vlora and was at the Tirana conference, where the rebellion was massive, but he managed to reduce the discontent and reverse the situation. The next step was to arrest the parties. Just as they were leaving the conference hall, not a few, but 44 communists were arrested and sentenced. Researcher Ana Lalaj writes that Enver Hoxha himself referred to the Tirana conference, describing it as one of the most challenging moments of the Socialist Party of Albania. ^17^

## Nikita Khruschev’s visit to Albania and the improvement of relations between the two parties during the years 1958–1959

Relations with the Soviet Union after Stalin’s death could never function independently of Yugoslavia’s relations with the Soviet Union. In a letter to the Central Committee of the Communist Party of the Soviet Union and specifically to Nikita Khrushchev on April 14, 1958, Enver Hoxha wrote enthusiastically about the decision taken by the Central Committee of the Communist Party of the Soviet Union not to send the Soviet delegation to the congress of the seventh of the League of Communists of Yugoslavia ^16^; “The Central Committee of the Albanian Labor Party is fully in solidarity with your decision on this issue. And he fully agrees with your Marxist-Leninist assessment of the draft program of the League of Communists of Yugoslavia”. ^16^ It was a direct quote since Enver Hoxha saw the relationship of the Soviet Union with Yugoslavia as a danger for the fading of bilateral cooperation between Albania and the Soviet Union. Enver Hoxha says:” Yugoslavia was an obstacle to realizing the projects and returning Albania as a strategic country in the Balkan Region. Soviet delegation not participating in the seventh congress of the Association of Communists of Yugoslavia enthused Enver Hoxha, and he expressed this in the letter he sent to Nikita Khrushchev on April 14, 1958. It raised Hoxha’s hope for further relations between the two countries Albania-Soviet Union. ^16^

The culmination of Albania’s relations with the Soviet Union, with too many analysts or witnesses of the time, was Nikita Khrushchev’s two-week visit to Albania. This visit lasted from May 25 to June 4 and was the first visit by such a senior Soviet leader. She seemed enthusiastic but also defiant. ^18^ The Prime Minister and Chairman of the Communist Party of the Soviet Union arrived in Tirana in May 1959 with a large contingent of Soviet officials. The Albanians enthusiastically received them, from high officials to the familiar people. During its stay in Albania, the Soviet delegation travelled throughout the country, visiting a range of industrial enterprises, agricultural cooperatives, state-owned agricultural enterprises, and research and teaching institutions. ^19^ In total, Khrushchev visited almost all the cities of Albania; in addition to Tirana, he went to Shkodra, Durres, Korca, Vlora, etc. Khrushchev’s visit to Albania marked a historical moment since the first time a senior leader of the Soviet Union had visited a small country like Albania. This achievement undoubtedly paid tribute to the party and its leader, Enver Hoxha, who proudly expressed the success of Khrushchev’s visit to Albania. In his book, Spartak Ngjela shows the moment like this: “I went out to see what to do with the school because all the schools in Tirana scattered along the entire road which started from Zogu I Zi and reached the Brigade Palace, where was Khrushchev’s two-week residence. There was frenzied enthusiasm everywhere. All the people shouted The Soviet Union and Enver-Khruschev’s names”. ^12^

During his visit to Albania, Nikita Khrushchev delivered a speech at a large rally with workers at the Stalin textile factory, a gift from the Soviet government to Albania. The history of the construction of this great work called Factory begins in the second five-year period of the Albanian communist government. The factory was built in 1948 and undertaken by the Soviet government. As for Khrushchev’s speech at the ‘Stalin’ combine, we will extract a short fragment where he openly threatens the Western countries by stating a thing. “You will build a missile base in Albania if one builds in Italy and Greece from the United States of America. ^20^ He said Albania was uniquely suited to build a missile base. The factory was Stalin’s gift to Albanians, so it became a “Stalin” textile factory. The textile factory “Stalin“was inaugurated by Enver Hoxha on November 8, 1951. It employed about 4,600 employees; each specialist and staff manager was trained in the Soviet Union. Radio Moscow reported that the Soviet leader’s car on the way to the combine was occasionally stopped by workers who wanted to hug the leader, the representative of a friendly country, and shake hands with him. ^20^

Despite this, Khrushchev’s visit to Albania in 1959 did not go well. Despite the enthusiasm, Khruschev’s visit to Albania brought a series of criticisms directed at the Albanian leader as a protest for the transparency of the investment of the donations that the Soviet Union had awarded to Albania. During his visit to Albania, Khruschev noticed a lot of poverty in the country, saying: “God has given this country all the opportunities and natural beauty to develop; you [referring to the Albanian citizens] should know the value.” Historians of the time also distinguished those disputes that existed despite statements that seemed to be on the same wavelength. ^21^^,^^22^ Only a year later, at the Moscow meeting in November 1960, Khrushchev was greatly surprised by the confrontation with angry Albanians. So, in Albania’s relations with the Soviet Union, the show had to be distinguished from the essence. In the central archive of the Albanian state, we found a series of top-secret (archive documents) about the time when Nikita Khrushchev’s remarks were listed during his visit to Albania. For instance, on May 25, 1959, Khrushchev said that in connection with the construction of the small metallurgical plant, one should first calculate how much a ton of metal would cost. Either for the construction of the new textile factory or the increase of the capacity of the ‘Stalin’ factory, the question must be whether other countries can accept the Albanian cotton that was poor quality and give good quality cotton in exchange. ^16^ Khrushchev constantly advised ensuring the math was done well, an area where Enver Hoxha’s power seemed to falter.

“Economy and agriculture are going well, and people are working. The country can become very rich and live without support. Albania has good development prospects because it has mines, good land, climate, and sea. We should not start from the fact that they grow here all cultures to meet the country’s needs but to cultivate those cultures for which there is more benefit”. ^16^

Khrushchev was fascinated by the Bay of Vlora during his visit to Albania. Enver Hoxha remembers this moment like this: “… .What a miracle is here. The ideal base could be built for our submarines. To excavate and throw these antiquities into the sea [they were talking about the archaeological objects of Butrint], let’s drill this mountain and get to the other side. We will have the most ideal and safest base in the Mediterranean. From this place, we can paralyze and attack everything. The Pasha Liman base was a pipe dream for the Soviets. More precisely, the dream of all Russian tsars was coming true. Their fleet would finally emerge in the Mediterranean. It would realize through Albania … .”. ^23^

## Findings

The findings in this paper are intended to be realized by analyzing data, and the realization of the study includes two years, 2018–2020. All the resources are provided for use in the Albanian language, where, without a doubt, the resources in foreign languages have been translated by the translation department within the State Archives. The number of documents is over 200 pages from the archives and over ten books referenced by Albanian and foreign authors.

The findings in this paper are intended to be realized by analyzing:

1. Features of the relationship between a small and weak country with a superpower.
2. these relations’ impact on our country’s internal and external context.
3. Ideological divergences in bilateral relations.
4. ‘Individual cults’ of leadership in both countries against the background of developing relations between the two countries.
5. The usefulness of Albania’s relations with the Soviet Union in these years.
6. Dependence on economic relations between Albania and the Soviet Union

In our research, we have used the state archive related to the relations between Albania and the Soviet Union. Enver Hoxha’s writings and some foreign or Albanian authors who were present at this time, giving their data and impressions, also helped our work. Based on this literature, we tried to bring an analysis of these relationships. Albania’s relations with the Soviet Union are still primarily undiscovered as long as many documents in the Russian archives are not available for study. However, like any study or work carried out in different periods, this one cannot set the last stone regarding these relationships. Maybe it could be an attempt to demystify or clarify something in this relationship with a lot of light and shadow. The documents of the Central State Archive of Albania do not give an impartial overview of the situation as they have been compiled with a load of ideological spirit and, therefore, do not present an accurate picture of the situation on both sites. Publications about Albanian-Soviet relations have been numerous in Albania but not in Russia. There are very few studies on this part of the history of the Soviet Union. The Russians have written a lot about relations with many countries of the world (not only with superpowers), but there is a marked lack of clarification of relations with our government.

The facts show that economic relations were precious. The value is explained by the fact that the financial aid that the Soviet Union gave to Albania led to the construction not only of infrastructure but also enterprises such as the ‘Stalin’ textile factory and many others like these, which led to the employment of thousands of workers and the increase to some extent in the standard of living Albania also took place in the international arena thanks to the contribution of the Soviet Union. There is no doubt that without their support, Albania was a bankrupt country, just as we emerged from friendship with Yugoslavia. Why was all this done? We hypothesize that this was because of Albania’s geographical position. The construction of the Pasha Liman naval base and the fascination of the Soviet leadership with the Gulf of Vlora indicated this. ^23^ However, the Soviets fled this country without much fuss, even though over the years, it was proclaimed in Albania that the Soviets quite coveted us because of their geostrategic goals. Pasha Liman’s base builds on an agreement and a highly secret document between the government of the People’s Republic of Albania and the Union of Soviet Socialist Republics. The document found in the archive of the Ministry of Foreign Affairs has the following content:

“… The Government of the People’s Republic of Albania and the government of the Union of Soviet Socialist Republics, to strengthen the defense of the People’s Republic of Albania, felt the need to conclude this agreement between them and purpose was appointed their plenipotentiaries: the chief of to the Albanian General Staff Major Arif Hasko and the Chief of the General Staff of the Soviet Naval Military Fleet Admiral Fokin V. A …”. ^3^ The agreement consisted of seven articles.

As for the de-Stalinization and liberalization of the country after the 20th Congress of the Communist Party of the USSR, which was dictated to Hoxha by the Soviet leader, history has shown that Hoxha maintained personal power at all costs. Tito’s Yugoslavia and the opinion that existed in the latter about Hoxha openly threatened Enver Hoxha and his power. The reasons for the split are manifold. On the one hand, in Yugoslavia, restoring Soviet-Yugoslav relations played a significant role in developing relations between Albania and the Soviet Union. However, we cannot say with conviction that Albania and the Soviet Union would have maintained their friendship if these relations had not been restored. But we have also seen this lineup in the light of the return of Soviet relations with Yugoslavia because exactly when it felt threatened by this return, Albania turned its eyes away from China and vice versa, and China found that there could be little support in Eastern Europe.

Considering that Enver Hoxha wanted to install the Stalinist regime in the country and keep it alive as long as possible to reach the desired conclusions, the world’s criticism of Stalinism must be carefully considered. Enver Hoxha’s desired conclusions were for Albania to maintain relations with the Soviet Union as long as possible for many reasons: economic, political, social, cultural, etc. At the same time, Enver Hoxha wanted to eliminate any relationship between Yugoslavia and the Soviet Union, as he saw it as a danger for Albania. So, restoring Yugoslavia-Soviet Union relations would mean the elimination of Albania in the diplomatic policy of the Eastern Bloc. Enver Hoxha claimed to be the only allied country of the Soviet Union in the Balkan region. Through theory and secondary documents, we manage to make a non-superficial study, but to unite the puzzle pieces, we must continue combining and clarifying different episodes with a common denominator.

Disputes within the communist bloc, such as those between Yugoslavia and the Soviet Union or Albania and Yugoslavia and the Soviet Union, showed that there could be significant divergences even when countries followed the same strategy and ideology. The cause of divergences in some cases has been sovereignty and essential issues of national character. As far as Albania is concerned, the facts show that the national interest ostensibly nurtured the ego and the struggle of Enver Hoxha for power. He used it face-to-face to maintain his power.

## Conclusions

Albania’s relations with the Soviet Union never emerged from the teaching of dependence and preferences. Although within the same ideological context, Albania’s relations with the Soviet Union reflected the relations of the powers and for this reason, they were always dominated by pressure, blackmail on the one hand, and bowing and persuasion on the other. Regional developments (relations between the USSR and Yugoslavia) harmed Albania’s relations with the Soviet Union. Communist ideology and the lack of democracy in Albania gave free rein to the communist leadership to maintain the monopoly of diplomacy by focusing on caste interests and communist interests.

The case of the analysis of Yugoslav influence in relations with the Soviet Union will continue to be a case study in international relations. About the Yugoslavs, Albania has never been a priority for the Soviet Union. In any case, it would have to be dealt with depending on the ups and downs of BS-Yugoslavia relations. This did not change in both Stalin and Khrushchev. Albania never managed to play in the field of international relations between the two greats.

In ideological assessment, as the Soviet Union embarked on the path of de-Stalinization and liberalization of the country after the XX Congress of the Communist Party of the USSR, a path dictated to Hoxha by the Soviet leader, history has shown that Hoxha retained power at all costs personal. The reasons for the split are manifold. On the one hand, Yugoslavia, the restoration of Soviet-Yugoslav relations, played a very important role in Albania’s relations with the Soviet Union. But we cannot say with conviction that Albania and the Soviet Union would have maintained their friendship if these relations had not been restored. Or if Albania would not have sided with China. But even this lineup in this paper I have seen in the light of the restoration of Soviet relations with Yugoslavia. Because exactly when it felt threatened by this return, Albania turned its eyes away from China and vice versa, China found that there could be little support in eastern Europe. Disputes within the communist bloc, such as those between Yugoslavia and the Soviet Union or Albania and Yugoslavia and the Soviet Union, showed that there could be significant divergences even when countries followed the same line and ideology. The cause of divergences in some cases has been sovereignty and essential issues of national character.

As far as Albania is concerned, the facts show that the national interest ostensibly nurtured the motto of International Relations. But further, numerous documents show that this could be the ‘best cover’ to safeguard the interests and personal power of the leadership. The study, regardless of the issues it addresses, does not aim to exhaust the topic of the paper but to contribute to the objective analysis and to have some impact on the continuation and deepening of the treatment of this topic. We live in a different time, but the phenomena are the same. The phenomena and challenges that the country faced in 1949–1959 are similar. Albania’s relations with the Soviet Union are still not fully revealed as long as many documents found in Russian archives are not available for study.

## Data availability

The underlying data for this study is under restriction by the Archival resources of the Albanian Central State Archives and the Central Archive of the Ministry of Foreign affairs. To request access to the same data used in this study, researchers should contact the information office within the premises of the Central Archives ( dpa@albarchive.gov.al). The employee of the Information Office orients the researcher about the documentation that must be submitted using archival resources in the internal environments of the archives. To access the archive and the use of resources in the interior, you must submit

1. A work certificate from the institution where you work.
2. A copy of the identity card.
3. A request describes the purpose of using the resources in the Archives, the topic, an explanation of the time frame for the completion of the study, as well as the contact email address or phone number.

Answers to requests are typically within ten days from the day of submission of the documentation according to the legal acts of the archives.
