# Supplementary material for: Analysis of Albania's diplomatic relations with the Soviet Union during the years 1948-1959
Source: F1000Res. 2024 Feb 9;11:1441. Originally published 2022 Dec 6. [Version 2] doi: 10.12688/f1000research.126497.2 (PMC10918309; doi:10.12688/f1000research.126497.2)
Supplement: Peer review history

# Reviewer report: Reviewer response for version 2

**DOI:** 10.5256/f1000research.162116.r245538

**Published:** 2024-03-06

**Authors:** Bekim Sejdiu

**Affiliations:** 1 Faculty of Law, University of Prishtina, Pristina, Kosovo (Serbia and Montenegro)

I hereby confirm that I approve the article.

Is the case presented with sufficient detail to be useful for teaching or other practitioners?

No

Is the work clearly and accurately presented and does it cite the current literature?

No

If applicable, is the statistical analysis and its interpretation appropriate?

Partly

Are all the source data underlying the results available to ensure full reproducibility?

Partly

Are the conclusions drawn adequately supported by the results?

No

Is the background of the case’s history and progression described in sufficient detail?

Partly

Reviewer Expertise:

NA

I confirm that I have read this submission and believe that I have an appropriate level of expertise to confirm that it is of an acceptable scientific standard.

# Reviewer report: Reviewer response for version 1

**DOI:** 10.5256/f1000research.138915.r230088

**Published:** 2024-01-30

**Authors:** Ardian Muhaj

**Affiliations:** 1 Institute of History, Tirana, Albania

**General evaluation:**

 The article defines sufficiently its research question or thesis. The authors effectively set the historical and geopolitical context. The article thoroughly covers the topic, including major events, key figures, and political dynamics. The sources cited are relevant and authoritative with a balanced mix of primary and secondary sources.

 The method used to analyze Albania's diplomatic relations with the Soviet Union are appropriate and well-executed. Also, the authors have clearly explained of how data and historical documents were collected and analyzed.

 The article analyzes effectively the diplomatic relations, and the arguments are well-supported with evidence. The discussion offers new insights on the subject. Overall, the historical information is presented accurately, and the article demonstrates a good understanding of the geopolitical dynamics of the period. The article contributes new perspectives to the field of diplomatic history or Albanian-Soviet relations.

**Specific suggestions:**

 Regarding **structure and clarity**, the article could have been better organized, allowing readers to follow the argument more easily. The language and style also need a thorough improvement.

 Regarding **references and citations**, the sources are properly cited, but the explanation of the content and the procedure to obtain the documents in the State Archive go into too much and unnecessary details.  Also, the bibliography should be enriched with more literature on the subject.

**Conclusion and Overall Evaluation:**

 The topic of the article is interesting, and the thesis statement is clear and sound.

 I recommend the article for indexing after the mentioned improvements have been made.

Is the case presented with sufficient detail to be useful for teaching or other practitioners?

Yes

Is the work clearly and accurately presented and does it cite the current literature?

Partly

If applicable, is the statistical analysis and its interpretation appropriate?

Not applicable

Are all the source data underlying the results available to ensure full reproducibility?

Yes

Are the conclusions drawn adequately supported by the results?

Yes

Is the background of the case’s history and progression described in sufficient detail?

Partly

Reviewer Expertise:

History of Albania; History of the Balkans; Economic History.

I confirm that I have read this submission and believe that I have an appropriate level of expertise to confirm that it is of an acceptable scientific standard.

# Reviewer report: Reviewer response for version 1

**DOI:** 10.5256/f1000research.138915.r181069

**Published:** 2023-08-02

**Authors:** Bekim Sejdiu

**Affiliations:** 1 Faculty of Law, University of Prishtina, Pristina, Kosovo (Serbia and Montenegro)

**General suggestion**

 The topic of the article is interesting and intriguing. The fact that the authors rely on archival documents adds to the value of the analysis.

 However, the Article needs profound revisions before publication.

 Two types of revisions need to be undertaken:

*First*, major language corrections and revisions need to be done as well as applying proper citation style and technical format.

*Second*, there should be a clear and consistent link between the stated research questions and hypothesis, with the analysis and findings. In the current draft, this is missing.

Some major comments are as follows:

**Abstract: **

- Abstract needs to be revised, to follow the usual format for Abstract.
- It is preferable to have explanations about the methodology, results, major argument/findings, in the Introduction, rather than in the Abstract.
- Not clear is the objective of this paper to highlight the attitude and intentions of the USSR approach towards Albania in this period? If yes, this needs to be stated clearly in the Abstract.
- It would be useful to add a sentence in the Abstract about the major argument of the paper?

**Introduction**

  

- Introduction has not been written in a proper/standardized form.
- Too much factual/descriptive details about Enver Hoxha in the Introduction.

**Hypothesis**

- Hypothesis need to be short, concise and in harmony with the explanation of the research questions.
- From the hypothesis, it transpires that Albania did not have any importance at all for the Soviet Union, but it was only a burden for Moscow. This is not easy to substantiate? Ideology and geography were intertwined, as a Cold War parameter. In this sense, it would be interesting to dig deeper in the interests of the Soviet Union in Albania. Khrushchev’s visit to Albania in 1959, which is elaborated in the paper, is a strong argument in this direction – particular the interpretation of this visit by Washington as an action intended to thwart the consolidation of nuclear superiority of Americans in the Southern Europe

**Overview of the history of Albania-Soviet Union relations**

- There is no consistency in the historical narrative: from the Ottoman-Russia war of the mid-XIX century, the authors jump to Lenin’s political visions and then to Enver Hoxha?
- Consistent analysis about relations between Albanian and Soviet Union is missing. 
- Furthermore, if the issue under discussing is history of “ Albania-Soviet U relations,” the focus needs to be in the Albanian state. This means providing at least a short overview of the position of Russia towards the first Albanian state of 1912/13; Ahmet Zogu’s era and then the Communist Albania (the latter is the major focus). 
- A clear distinction should be made between the heavy element of propaganda in the Albanian communist historiography – which sought to glorify the ostensibly deep historical ties with Russia –  with the real historical facts.

**Yugoslav factor**

- It is undisputable that Yugoslav communist party (with emissaries such as Serbian communist Dusan Mugosa and Miladin Popovic), played crucial role in creating and leading from behind, the Albanian Communist Party and, from 1045 – 1948, Albania was a satellite of Belgrade.
- It would be interesting to explore briefly, why Enver Hoxha broke with Tito in 1948, and turned robustly towards Moscow (Albanian Communist state was an ally of
- Belgrade, even in suppressing anti-Yugoslav uprising of Albanians in Kosovo, in 1945, lead by Shaban Polluzha)?

**Results**

- No consistency between the analysis in the preceding section and the results?
- There needs to be a stronger and more direct link between the research questions; major hypothesis and the analytical part of the paper. This linkage is very weak, not to say non-existing.
- In this vein, the economic and political dependency of Albania from the Soviet Union needs to be clearly and substantially argued.
- Further, how it is argued in this paper that: “The Soviet Union did not have the geopolitical or economic interest in Albania? This is the first hypothesis?
- Or, how does this article address the research question: “Was it possible for Enver Hoxha to behave differently with the Soviet Union and what has been Albania’s strong point in relations with the Soviet Union”?

**Language and technical problems in the text**

 The draft needs thorough revision, with regards to the language editing, style of citation and technical aspects. 

- Many sentences are unclear, not properly formulated and confusing.
- Beginning of new, important arguments, to be done in a new sentence with one empty space in between.
- Space between new arguments: not consistent.
- Citation style and the titles of the sections: to have a standard format.
- Titles of the parts do not follow any standard format.
- The part on methodology, comparative research, limitation, would be OK for a book, but not for an academic journal?

Is the case presented with sufficient detail to be useful for teaching or other practitioners?

No

Is the work clearly and accurately presented and does it cite the current literature?

No

If applicable, is the statistical analysis and its interpretation appropriate?

Partly

Are all the source data underlying the results available to ensure full reproducibility?

Partly

Are the conclusions drawn adequately supported by the results?

No

Is the background of the case’s history and progression described in sufficient detail?

Partly

Reviewer Expertise:

NA

I confirm that I have read this submission and believe that I have an appropriate level of expertise to state that I do not consider it to be of an acceptable scientific standard, for reasons outlined above.

# Response

**Published:** 2023-08-06

**Authors:** mirela tase

These are some comments relating to the review`s report as follows:

 We want to clarify that the length or brevity of an argument does not affect the construction of hypotheses, despite what the reviewer may have suggested. We have developed three hypotheses that fully align with our research questions. Each hypothesis is thoroughly explained, using both research sources and our well-articulated ideas to establish the significance of the relationships between both sides. As evidence, we can provide the following quote:

 “We tried, through hypotheses, to provide an assessment and undoubtedly a clearer insight into the relations with the Soviet Union. According to our judgment, Albania was an unimportant country for the Soviet Union (due to the way Enver...) Enver Hoxha benefited from this relation (with the goal of building...) the socialist state and giving it a name in the international arena, not only for himself (head of Albania), but also Albania as a whole.”

 While we appreciate the reviewer's argument about Khrushchev's visit to Albania in 1959, which suggests that Washington saw the visit as a move to prevent American nuclear superiority in Southern Europe, we must clarify that this argument is not directly related to the theme of our paper, or the three hypotheses presented. However, we will consider it a suggestion to explore in a separate article that addresses this issue.

 In regard to the Overview of the history of Albania-Soviet Union relations, the reviewer suggested including a brief overview of Russia's position towards the first Albanian state in 1912/13. However, this is irrelevant to our paper's primary focus, which is on the period from 1945 onwards. This suggestion is not directly related to the paper's topic. However, we will try to integrate it into another work that focuses on Albania's relations with BS in the beginnings of the Albanian state and in continuity.

 Regarding the reviewer's suggestions, we would like to clarify that the connection between the research questions, main hypothesis, and analytical part is not weak but very strong and visible throughout the paper. We emphasized multiple times in the work's results and description that the Soviet Union had no direct political or economic interests in Albania. However, they provided financial assistance during both the Stalin and Khrushchev periods, which benefited Albania's construction of a socialist state. Despite the lack of direct interest from the Soviet Union, the paper provides a clear argument for Albania's economic and political dependence on them, which is also the focus of Enver Hoxha's relations with BS.

 Regarding the answer to the unexplained question, according to the reviewer, "Was it possible for Enver Hoxha to behave differently with the Soviet Union, and what was Albania's strong point in relations with the Soviet Union," this was very clearly addressed in the paper and mainly in the results. This is included in the conclusion as follows. "In conclusion, I would summarize with the expression, “Stalin was the political muse for Enver Hoxha, this worship that he had continued until the end of his life and refused to be de-Stalinized. This turned out to be fatal in relations with the Soviet Union and all other countries and ended in the greatest

 isolation of Albania until the fall of the regime in 1990.” Albania's strong point in Albania-BS relations was Hurshov’s several-day visit to Albania, where he ranked our country as one of the most influential countries and Albania's geopolitical position in the Balkans."

 Following the answer "Enver Hoxha, if he did not prioritize the longevity of his power and his jealousy towards the relations between BS and Yugoslavia, where he saw the latter as a danger to the end of his relations with BS, he would have no only a vital place in these bilateral relations, but it would give a different look to the relations between these two countries. Enver Hoxha's insistence on preserving the Stalinist cult at all costs, and (his rejection of the criticisms made by  (Khrushchev) to Stalin clearly show his fear of reform as the most significant risk of leaving power.

 Albania's strong point in Albania-BS relations was a several-day visit to Albania, where he ranked our country as one of the most influential countries and Albania's geopolitical position in the Balkans.

 In final publishing, we will edit the Language by the English editor.

 Best wishes 

 Authors
